# Methane Saline Ameliorates Traumatic Brain Injury through Anti-Inflammatory, Antiapoptotic, and Antioxidative Effects by Activating the Wnt Signalling Pathway

**DOI:** 10.1155/2020/3852450

**Published:** 2020-12-17

**Authors:** Meng Li, Weiman Gao, Le Ji, Jia Li, Wanting Jiang, Wenchen Ji

**Affiliations:** ^1^Department of Orthopedics, The First Affiliated Hospital of Xi'an Jiaotong University, 710061, China; ^2^Department of Orthopedics, Shaanxi Provincial People's Hospital, 710068, China; ^3^Department of Ultrasound Diagnosis, Xi'an People's Hospital (The Fourth Hospital of Xi'an), 710004, China

## Abstract

**Objective:**

Methane saline (MS) can be used to treat many diseases via its anti-inflammatory, antiapoptotic, and antioxidative activities. However, to date, there is no published evidence as to whether MS has any effect on traumatic brain injury (TBI). The Wnt signalling pathway regulates cell proliferation, differentiation, migration, and apoptosis; however, whether the Wnt signalling pathway regulates any effect of MS on TBI is unknown. This study was designed to explore the role of MS in the treatment of TBI and whether the Wnt pathway is involved.

**Methods:**

Sprague-Dawley rats were randomly divided into five groups: sham, TBI, TBI+10 ml/kg MS, TBI+20 ml/kg MS, and TBI+30 ml/kg MS. After induction of TBI, MS was injected intraperitoneally once daily for seven consecutive days. Neurological function was evaluated by the Neurological Severity Score (NSS) at 1, 7, and 14 days after TBI. Haematoxylin-eosin (HE) staining, inflammatory factors, neuron-specific enolase (NSE) staining, oxidative stress, and cell apoptosis were measured and compared 14 d after TBI to identify the optimal dose of MS and to investigate the effect of MS on TBI. In the second experiment, Sprague-Dawley rats were randomly divided into four groups: sham, TBI, TBI+20 ml/kg MS, and TBI+20 ml/kg MS+Dickkopf-1 (DKK-1, a specific inhibitor of the Wnt pathway). NSE, caspase-3, superoxide dismutase (SOD), Wnt3a, and *β*-catenin were detected by real-time PCR and Western blotting. The results from each group were compared 14 d after TBI to determine the regulatory role of the Wnt pathway.

**Results:**

Methane saline significantly inhibited inflammation, oxidative stress, and cell apoptosis, thus protecting neurons within 14 days of TBI. The best treatment effect against TBI was obtained with 20 ml/kg MS. When the Wnt pathway was inhibited, the treatment effect of MS was impaired.

**Conclusion:**

Methane saline ameliorates TBI through its anti-inflammatory, antiapoptotic, and antioxidative effects via activation of the Wnt signalling pathway, which plays a part but is not the only mechanism underlying the effects of MS. Thus, MS may be a novel strategy for treating TBI.

## 1. Introductions

Traumatic brain injury (TBI) is a common disease that is primarily caused by car accidents and high-altitude falls. It can result in neurological dysfunction and death. TBI has two clinical phases: primary and secondary injury [1]. The former involves deformation of the brain caused by a direct force; once this happens, it is irreversible [2]. The latter phase is the cascade of responses that occur several hours after the primary injury, among which inflammatory responses and oxidative stress are the main factors underlying the local microenvironmental disorder [3]. Alleviation of these responses is essential for a functional recovery.

Methane has been extensively studied ever since its discovery in 1778 [4]. It was previously thought that highly concentrated methane (methane/air volume > 30%) caused poisoning while low concentrations have no physiological effects [5, 6]. However, recently, it has been shown that methane has biological properties, especially anti-inflammation, antioxidative, and antiapoptotic effects and can be used to treat acute lung injury, autoimmune hepatitis, and retinal ischaemia injury [7–9]. The treatment mechanisms appear to be related to the regulation of mitochondrial function and proteins involved in signalling pathways [10, 11]. Given the volatility of methane, methane saline (MS), which is prepared under high-pressure conditions and has similar properties to methane, is used for treatment and research due to its ease of transport. However, it is not known whether MS can treat TBI and what signalling pathways are involved in the potential effects of MS on TBI. The Wnt pathway plays an important antiapoptotic role, maintains neuron survival, and modulates antioxidative stress [12]. Here, we determined whether MS has a therapeutic effect on TBI and the role of the Wnt pathway. This is the first time that MS has been used to treat TBI and that the regulatory mechanism has been described, thus building a theoretical basis for clinical use of MS.

## 2. Materials and Methods

### 2.1. Animals

Sprague-Dawley (SD) rats weighing 250–300 g were provided by the Animal Experimental Centre of the Xi'an Jiaotong University Health Care Centre. Animals were kept in a suitable environment with sufficient water and food. Animal experiments were approved by the Ethics Committee of the Xi'an Jiaotong University Health Care Centre.

### 2.2. Methane Saline Preparation

Methane saline was provided by the hepatobiliary surgery department of the Xi'an Jiaotong University 1st Affiliated Hospital. The method for producing MS has been described previously [13]. MS was prepared 24 h before injection to ensure a high concentration. After adding methane (purity > 99.999%) to saline, the solution was placed in a high-pressure (0.4 MPa) device (Wuhan Newradar Special Gas Co., Ltd., Wuhan, China) for 4 h. The MS (0.99 mmo1/l) was stored at 4°C for further use.

### 2.3. Preparation of Rat Traumatic Brain Injury Model

After administration of anaesthesia (chloral hydrate, 400 mg/kg, ip), rats were fixed on an operation table and a 5 mm diameter hole was drilled 1 mm from the right coronal suture and 2 mm from the sagittal suture without damaging the underlying dura. An improved Feeney free fall device was adopted, which used a 40 g hammer falling from a height of 25 cm, causing a 5 mm depth impact. Penicillin (400,000 u/d) was injected postoperatively for three consecutive days to prevent infection.

### 2.4. Experimental Design and Protocols

The experiment was divided into two parts. The first part was performed to determine the optimal dose of MS and to explore the mechanisms underlying the effects of MS. Rats were divided into five groups with 10 SD rats in each group (Supplement 1). For the sham group, the incision was sutured immediately after the hole was drilled (without impact). For the TBI group, no treatment was given after the TBI impact. The TBI+10 ml/kg MS, TBI+20 ml/kg MS, and TBI+30 ml/kg MS groups received the corresponding doses of MS once a day for seven days.

The second part of the experiment was performed to determine the role of the Wnt pathway in the effects of MS on TBI. DKK-1 is an antagonist of the Wnt pathway. It was dissolved in sterile PBS, and the concentration was controlled at 1 g/l. Rats were divided into four groups, with 10 SD rats in each group (Supplement 2). The sham group, TBI group, and TBI+20 ml/kg group were the same as described in the first part of the experiment. The TBI+20 ml/kg MS+DKK-1 group received a 1 *μ*l injection of DKK-1 into the encephalocele 30 min before surgery, and then 20 ml/kg MS was administered once per day for seven days after surgery. The DKK-1 injection speed was 1 *μ*l/min. The encephalocele was localised as follows: 0.8 mm behind the fontanelle, with a diameter of 1.5 mm and at a depth of 3.5 mm.

### 2.5. Neurological, Morphological, and Inflammatory Factors

On the 1^st^, 7^th^, and 14^th^ postoperative days, the Neurological Severity Score (NSS) of each rat was evaluated by two nonresearchers. The maximum NSS is 18, including the exercise test, sensory test, balance test, and reflex test. Higher scores represent severe neurological damage [14]. Fourteen days after surgery, the rats were sacrificed by cervical dislocation (*N* = 10). Then, the brains were completely removed (*N* = 6), and cerebral cortex tissue around the impact area (width 2 mm, length 3 mm, and thickness 3 mm) was removed and fixed with 4% paraformaldehyde for two days and then coated in paraffin. The tissue was sliced into 5 *μ*m thick sections for HE staining, NSE staining (Abcam, USA), caspase-3 staining (Abcam), and SOD staining (Abcam). The staining steps were as follows: after deparaffinization, sections were treated with 3% hydrogen peroxide for 15 min to inhibit endogenous peroxidase activity. Antigen retrieval was then performed using 0.01 M citrate buffer (pH 6.0) at 85°C for 12 min. Sections were preincubated with normal goat serum for 20 min at room temperature, prior to incubation at 4°C overnight with the above antibodies. Sections were then incubated with secondary antibodies.

The brains of the remaining rats (*N* = 4) were removed, homogenized on ice with PBS, and then centrifuged at 4°C and 10,000 rpm for 30 min to collect the supernatant for ELISA to detect inflammatory cytokines, including IL-1*β* (Abcam), IL-6 (Abcam), and TNF-*α* (Abcam). The operation method was carried out according to the manual of the ELISA kit.

### 2.6. Measurement of Staining

After HE staining, the morphology of the red cytoplasm and the blue nucleus and the inflammatory cells could be observed. The expression of SOD, NSE, and caspase-3 was immunohistochemically detected as brown particles in the cytoplasm. Highly expressed areas were identified under a low-power lens (×200), while cell morphology was observed under a high-power lens (×400).

Six sections were selected for SOD, and five different overlapping fields were randomly selected in each section to examine the integrated optical density (IOD) and positive cell area of SOD. Mean IOD (MOD) was used for statistical analysis [MOD = sum(IOD)/sum(area)]. NSE and caspase-3 were evaluated by positive cell counts. Six sections were taken from each group, and five different overlapping fields were randomly selected in each section to count the positive cells for statistical analysis.

### 2.7. Detection of mRNA Expression by Real-Time PCR

On the 14^th^ postoperative day, brain sections (6 × 4 × 3 mm) containing the centre of the impact point were obtained from the SD rats in each group (*N* = 5). Total RNA was isolated from the brain tissue, and reverse transcription of the purified RNA was performed using oligo(dT) priming according to the manufacturer's instructions (TaKaRa, Japan). Real-time PCR was performed using SYBR green. The primer pairs for NSE, caspase-3, SOD, Wnt3a, *β*-catenin, and *β*-actin, which served as the housekeeping gene, are listed in Table 1 (Sangon Biotech, China). The relative gene expression was obtained from the standard curve using the 2^-*ΔΔ*Ct^ method [15].

### 2.8. Western Blotting

On the 14^th^ postoperative day, brain sections (6 × 4 × 3 mm) containing the centre of the impact point were obtained from the SD rats in each group (*N* = 5). RIPA lysis buffer was used to extract protein from the brain. Protein samples were electrophoresed using 10% SDS-PAGE and transferred to PVDF membranes (Abcam). Nonspecific reactivity was blocked with 5% skimmed milk in Tris-buffered saline containing Tween-20 for 60 min at room temperature. Membranes were incubated with rabbit anti-rat NSE, caspase-3, SOD, Wnt 3a (Abcam), and *β*-catenin (Abcam) antibodies at 4°C overnight, followed by incubation with goat anti-rabbit antibodies (Abcam). *β*-Actin antibodies (Abcam) were used as an internal control. The relative amounts of proteins were measured using Image Lab 8.0 (Media Cybernetics, USA).

### 2.9. Statistical Analysis

The data for each group were analysed by SPSS 18.0 (IBM, USA) and are expressed as mean ± standard deviation. Neurological function was evaluated by repeated-measure ANOVA and one-way ANOVA. Between-group differences in the other indicators were analysed by one-way ANOVA. *P* < 0.05 was considered to be statistically significant.

## 3. Results

### 3.1. Neurological Function Tests

**Figure 1:**
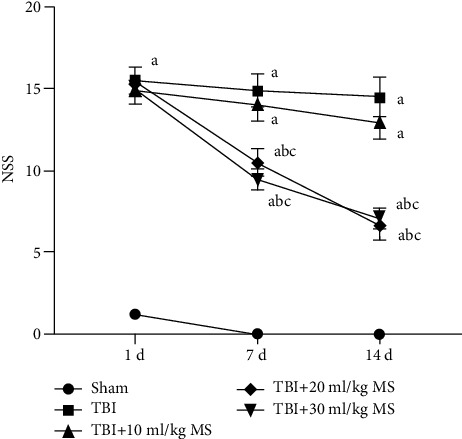
Neurological function scores at different points in each group. The data are expressed as the mean ± SEM. Repeated ANOVA was used for NSS score trend comparison. One-way ANOVA was used for group comparison at the same time point. ^a^*P* < 0.05, compared with the sham group as determined by one-way ANOVA. ^b^*P* < 0.05, compared with the TBI group as determined by one-way ANOVA. ^c^*P* < 0.05, compared with the TBI+10 ml/kg MS group as determined by one-way ANOVA.

The TBI, 10 ml/kg MS, 20 ml/kg MS, and 30 ml/kg MS groups exhibited severe neurological damage with significantly higher NSS scores than the sham group (*P* < 0.05). One day after the operation, there were no statistically significant differences in the NSS scores between the TBI, 10 ml/kg MS, 20 ml/kg MS, and 30 ml/kg MS groups. The NSS scores of the TBI, 10 ml/kg MS, 20 ml/kg MS, and 30 ml/kg groups exhibited a downward trend on the 7^th^ and 14^th^ postoperative days compared to scores on the 1^st^ postoperative day. However, the 20 and 30 ml/kg MS groups were the only groups with statistically significant reductions in NSS scores (*P* < 0.05). This indicates that injection of 20 ml/kg MS for seven days may help recover neural function following TBI to some extent. However, a further increase in the dosage of MS to 30 ml/kg produced no additional recovery of NSS function (Figure 1).

### 3.2. HE Staining

**Figure 2:**
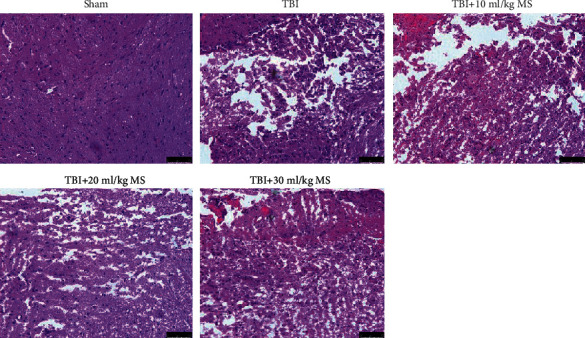
HE staining in each group (×200).

In the sham group, the brain tissue exhibited normal physiological features with a large number of normal neurons. The TBI group and the 10 ml/kg MS groups exhibited necrosis and oedema in the brain tissue with infiltration of a large number of inflammatory cells. The 20 ml/kg and 30 ml/kg MS groups exhibited milder damage than the TBI and 10 ml/kg MS groups. The brain structure in the 20 ml/kg MS group was the best with only minor inflammation (Figure 2).

### 3.3. Inflammatory Factors

**Figure 3:**
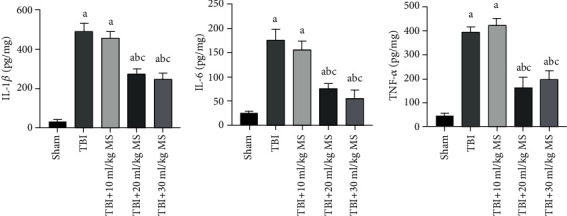
Inflammatory factors and statistical results. The data are expressed as the mean ± SEM. ^a^*P* < 0.05, compared with the sham group as determined by one-way ANOVA. ^b^*P* < 0.05, compared with the TBI group as determined by one-way ANOVA. ^c^*P* < 0.05, compared with the TBI+10 ml/kg MS group as determined by one-way ANOVA.

Levels of IL-1*β*, IL-6, and TNF-*α* were dramatically higher in the TBI, 10 ml/kg MS, 20 ml/kg MS, and 30 ml/kg MS groups compared with the sham group (*P* < 0.05). Although there were no statistically significant differences in the levels of the inflammatory factors between the TBI and 10 ml/kg MS groups, the level of inflammation in these two groups was significantly higher than that observed in the 20 and 30 ml/kg MS groups (*P* < 0.05). Further, there was no difference between the 20 and 30 ml/kg MS groups in terms of inflammatory factors (Figure 3). These data indicate that 20 ml/kg MS can significantly inhibit the release of inflammatory factors, and this treatment effect is not further improved by increasing the MS dose to 30 ml/kg.

### 3.4. NSE Staining

**Figure 4:**
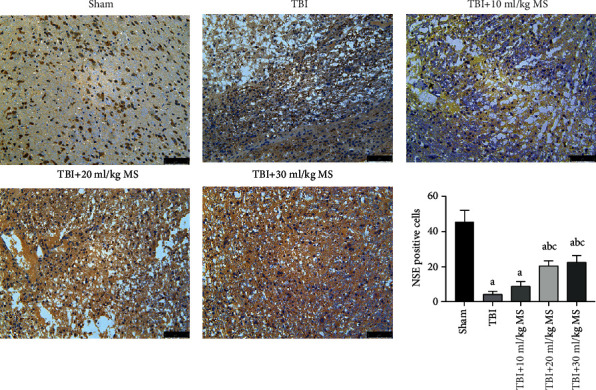
NSE staining (×200 magnification) in each group and statistical results. The data are expressed as the mean ± SEM. ^a^*P* < 0.05, compared with the sham group as determined by one-way ANOVA. ^b^*P* < 0.05, compared with the TBI group as determined by one-way ANOVA. ^c^*P* < 0.05, compared with the TBI+10 ml/kg MS group as determined by one-way ANOVA.

Normal neurons and positive NSE staining were observed in the sham group. There was no significant difference between the 20 and 30 ml/kg MS groups (*P* < 0.05). However, the numbers of NSE-positive cells in the 20 and 30 ml/kg groups were significantly higher than those in the TBI and 10 ml/kg MS groups (*P* < 0.05). These results indicate that MS has a neuroprotective effect at a dose of 20 ml/kg, and this effect is not enhanced when the dose is increased to 30 ml/kg (Figure 4).

### 3.5. Caspase-3 Staining

**Figure 5:**
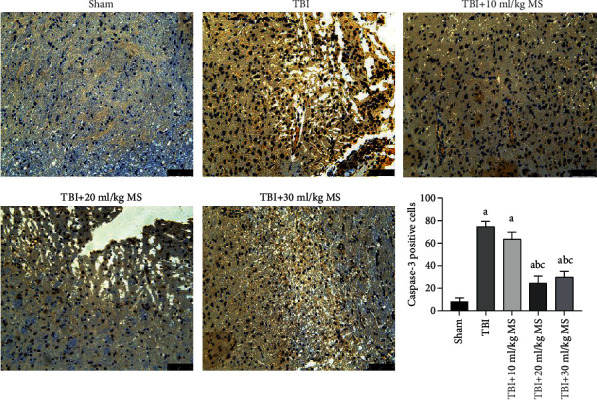
Caspase-3 staining (×200 magnification) in each group and statistical results. The data are expressed as the mean ± SEM. ^a^*P* < 0.05, compared with the sham group as determined by one-way ANOVA. ^b^*P* < 0.05, compared with the TBI group as determined by one-way ANOVA. ^c^*P* < 0.05, compared with the TBI+10 ml/kg MS group as determined by one-way ANOVA.

There were a few brown apoptotic cells observed in the sham group, which is a normal physiological phenomenon. There was no statically significant difference between the 20 and 30 ml/kg MS groups in terms of caspase-3-positive cells (*P* < 0.05). There were fewer caspase-3-positive cells in the 20 and 30 ml/kg MS groups than in the TBI and 10 ml/kg MS groups (*P* < 0.05, Figure 5). This indicates an antiapoptotic effect of MS that is optimal at a dose of 20 ml/kg. There was no improvement in the antiapoptotic effect by increasing the dose to 30 ml/kg.

### 3.6. SOD Staining

**Figure 6:**
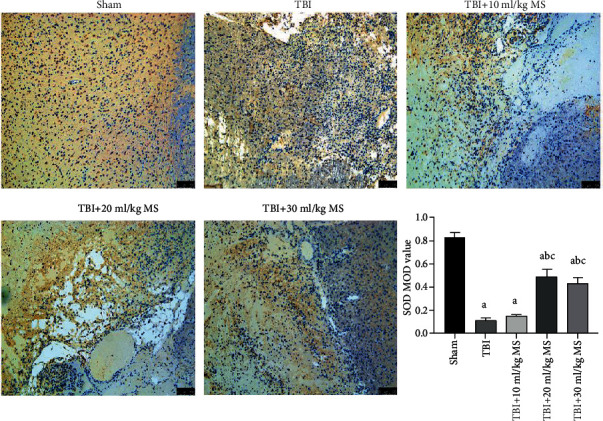
SOD staining (×200 magnification) in each group and statistical results. The data are expressed as the mean ± SEM. ^a^*P* < 0.05, compared with the sham group as determined by one-way ANOVA. ^b^*P* < 0.05, compared with the TBI group as determined by one-way ANOVA. ^c^*P* < 0.05, compared with the TBI+10 ml/kg MS group as determined by one-way ANOVA.

The SOD values of the TBI, 10 ml/kg MS, 20 ml/kg MS, and 30 ml/kg MS groups were significantly lower than that of the sham group (*P* < 0.05). The 20 and 30 ml/kg MS groups had higher SOD values than the TBI and 10 ml/kg MS groups, but there was no statistically significant difference in the SOD values of the 20 ml/kg MS and 30 ml/kg MS groups (Figure 6). This indicates that 20 ml/kg MS had the strongest antioxidative effect and increasing the dose to 30 ml/kg does not improve the antioxidant effect.

### 3.7. Detection of mRNA and Protein Expression by Real-Time PCR and Western Blot

**Figure 7:**
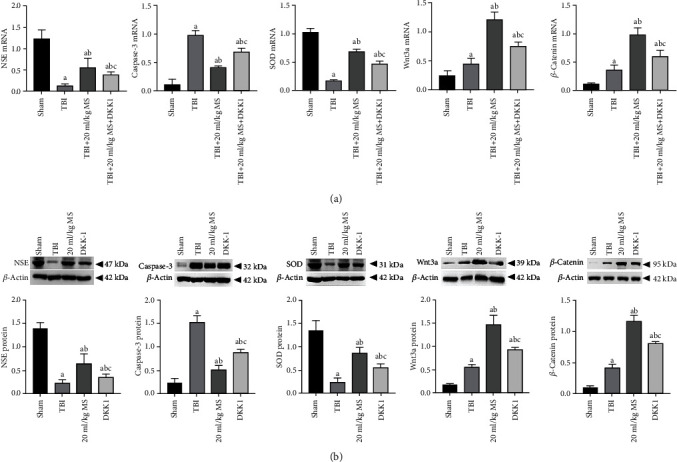
mRNA and protein expression in each group and statistical results. The data are expressed as the mean ± SEM. ^a^*P* < 0.05, compared with the sham group as determined by one-way ANOVA. ^b^*P* < 0.05, compared with the TBI group as determined by one-way ANOVA. ^c^*P* < 0.05, compared with the TBI+20 ml/kg MS group as determined by one-way ANOVA.

In order to clarify the signalling pathways that are involved in the therapeutic effect of MS on TBI, we performed real-time PCR after 14 days (Figure 7(a)). Compared with the 20 ml/kg group, the DKK-1 group had statistically significantly lower NSE, SOD, Wnt3a, and *β*-catenin expressions and higher caspase-3 expression (*P* < 0.05). However, when compared with the TBI group, the expressions of NSE, SOD, Wnt3a, and *β*-catenin in the DKK-1 group were increased while the expression of caspase-3 was decreased (*P* < 0.05). These results indicate that the therapeutic effect of 20 ml/kg MS on TBI involves the inhibition of apoptosis, the maintenance of physiological neuronal structure, and antioxidative effects. However, after adding DKK-1, a Wnt pathway inhibitor, the above effects were weakened and TBI was aggravated. The Western blot results were consistent with the real-time-PCR results (Figure 7(b)).

## 4. Discussion

Traumatic brain injury, a clinical disease with a 30% mortality rate, brings a heavy economic burden to families and society [16]. After TBI, inflammatory factors such as TNF-*α*, IL-1, and IL-6 are secreted and can induce oxidative stress responses and apoptosis, resulting in blood-brain barrier (BBB) impairment and cerebral oedema [17–19]. Though mild inflammation can protect the body, excessive inflammatory reactions aggravate the injury [20]. Therefore, inhibition of inflammation and oxidation plays an important role in neurological recovery after TBI, and new treatment methods must be explored. Wang and colleagues treated spinal cord injury with MS and found that MS remains in the spinal cord for 72 hours after only 10 minutes of perfusion, which demonstrates good biocompatibility and penetration, thus making it suitable for the treatment of diseases in the central nervous system [21]. However, to date, there have been no reports on the therapeutic effect of MS on TBI.

The first part of this study was performed to determine whether MS has a role in the treatment of TBI and, if so, its optimal dose. Different doses (10, 20, and 30 ml/kg MS) were used to treat TBI for seven consecutive days after surgery. On the 14^th^ day after the operation, brain tissue morphology, inflammatory factors, caspase-3, NSE, and SOD expression in the 10 ml/kg MS group were not significantly different from those in the TBI group. However, increased NSS, more residual neurons, and higher SOD were observed while reduced expression of TNF-*α*, IL-1, and IL-6 and decreased apoptosis were observed in the 20 ml/kg and 30 ml/kg MS groups. These results indicate that MS can inhibit inflammation and apoptosis in brain tissue, protect brain morphology, and improve nerve function after injury and that the effective dose of MS for treating TBI is 20 ml/kg. When the MS dose was increased to 30 ml/kg, there was no further improvement in the therapeutic effect. In fact, as the rats are relatively small, injection of excessive fluid can produce a heavy load on the heart. Therefore, we believe that 20 ml/kg MS is the optimal dose for treating TBI. Moreover, we found that the outcomes of the 20 ml/kg group were different from those of the sham group (repair effect was far from the sham group), which indicates that TBI secondary injury involves a series of complex pathological processes. Anti-inflammation and inhibition of oxidative stress and apoptosis can only achieve partial treatment effects. A complete cure requires a comprehensive understanding of the signalling pathways involved in the therapeutic mechanism.

Based on this, we designed the second part of the experiment. The Wnt signalling pathway regulates cell proliferation, differentiation, migration, and apoptosis [22]. Wnt3a is an important member of the Wnt family and can bind to receptors on the cell membrane to inhibit the degradation of the APC/Axin/GSK-3*β* complex and promote the accumulation of *β*-catenin in the cytoplasm, thereby activating the expression of target genes and regulating the cell cycle [23]. Wnt3a expression increases when the Wnt signalling pathway is activated and *vice versa*, and thus, the level of Wnt3a can directly reflect the status of the Wnt pathway [24]. To explore whether the Wnt pathway plays a role in the treatment effect of MS in TBI, we preinjected DKK-1 (a specific inhibitor of the Wnt pathway that can block the binding between Wnt proteins and cell membrane receptors) into the encephalocele of rats. The DKK-1 group had lower NSE, SOD, Wnt3a, and *β*-catenin but higher caspase-3 compared with the 20 ml/kg MS group. Moreover, when compared to the TBI group, the DKK-1 group had higher NSE, SOD, Wnt3a, and *β*-catenin but lower caspase-3. These results indicate that the Wnt pathway is involved in the treatment effect of MS on TBI via anti-inflammatory, antioxidative, and antiapoptotic activities, but inhibiting the Wnt pathway does not completely eliminate the therapeutic effect of MS, highlighting the possibility that other signalling pathways are involved. Further research is currently underway to identify other related pathways in order to improve the outcomes of TBI.

The novelty of this study is the use of MS in the treatment of TBI and the description of the mechanism of action and the role of the Wnt signalling pathway in the treatment process. However, the current study has certain limitations, such as a lack of long-term results. This research was conducted in the early and middle stages of TBI, and thus, further experiments should focus on the later stages of TBI. In addition, the effective dose (20 mg/kg MS) in this study was consistent with the results of Wang et al. for the treatment of spinal cord injury but differed from the results of Fan et al. (10 mg/kg) [25]. The difference may be due to the use of different animal models and administration frequencies. In our experiment, the TBI model and once-daily administration of MS for seven consecutive days were adopted while Fan et al. used models of CO poison and administration every 8 h (0, 8, and 16 h).

In conclusion, MS can effectively contribute to TBI recovery, possibly due to activation of the Wnt pathway and its anti-inflammatory, antioxidative, and antiapoptotic activities. These therapeutic mechanisms may provide a foundation for the clinical application of MS in the treatment of TBI, thereby improving the outcomes of TBI patients.

## Figures and Tables

**Table 1:** Primer sequences.

Name of primer	Primer sequences(5′-3′)
NSE	F-AGCGTTACTTAGGGCAAAGGTGTCC
	R-AACTCCAGCATCAGGTTGTCCAG
Caspase-3	F-CTGGACTGCGGTATTGAGAC
	R-CCGGGTGCGGTAGAGTAAGC
SOD	F-TGGTGTGGCCGATGTGTCTA
	R-TCCAGCGTTTCCTGTCTTTG
Wnt3a	F-GAATGGTCTCTCGGGAGTTTGC
	R-CAGCAGGTCTTCACTTCGCAAC
*β*-Catenin	F-GACAAGCCACAGGACTACAAGAA
	R-CGTATCCACCAGAGTGAAAAGAA
*β*-Actin	F-CGGGAAATCGTGCGTGAC
	R-TGGAAGGTGGACAGCGAGG

## Data Availability

The data sets supporting the conclusions of this article are included within the article and available from the corresponding author on reasonable request.

## Additional Points

*Statement of Human and Animal Rights*. The experimental procedures in this study were conducted in accordance with the Ethics Committee of Xi'an Jiaotong University's approved protocols.

## Supplementary Materials

Supplementary Materials Supplement 1: first part—experimental protocol. Supplement 2: secondary part—experimental protocol.
