# Correction to “Methane Saline Ameliorates Traumatic Brain Injury Through Anti‐Inflammatory, Antiapoptotic, and Antioxidative Effects by Activating the Wnt Signalling Pathway”

**DOI:** 10.1155/bmri/9827521

**Published:** 2026-04-06

M. Li, W. Gao, L. Ji, J. Li, W. Jiang, and W. Ji, “Methane Saline Ameliorates Traumatic Brain Injury Through Anti‐Inflammatory, Antiapoptotic, and Antioxidative Effects by Activating the Wnt Signalling Pathway,” *BioMed Research International* 2020, no. 1 (2020): 3852450, https://doi.org/10.1155/2020/3852450.

In the article titled “Methane Saline Ameliorates Traumatic Brain Injury Through Anti‐Inflammatory, Antiapoptotic, and Antioxidative Effects by Activating the Wnt Signalling Pathway” there was an error in Figure 5. More specifically, the panel ‘TBI+10 ml/Kg’ showed an image depicting the ‘TBI’ group. The error occurred during figure assembly and the correct Figure 5 is shown below:

**Figure 5:**
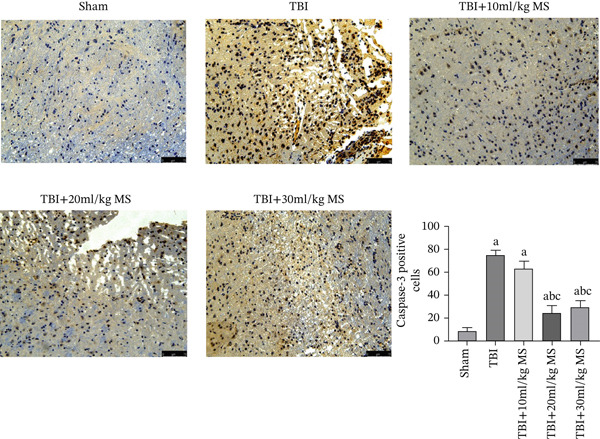
Caspase‐3 staining (×200 magnification) in each group and statistical results. The data are expressed as the mean ± SEM. ^a^ *p* < 0.05, compared with the sham group as determined by one‐way ANOVA. ^b^ *p* < 0.05, compared with the TBI group as determined by one‐way ANOVA. ^c^ *p* < 0.05, compared with the TBI+10 mL/kg MS group as determined by one‐way ANOVA.

We apologize for this error.

