# Supplementary material for: Methane Saline Ameliorates Traumatic Brain Injury through Anti-Inflammatory, Antiapoptotic, and Antioxidative Effects by Activating the Wnt Signalling Pathway
Source: Biomed Res Int. 2020 Dec 17;2020:3852450. doi: 10.1155/2020/3852450 (PMC7762637; doi:10.1155/2020/3852450)

Supplement 1 First part Experimental protocol


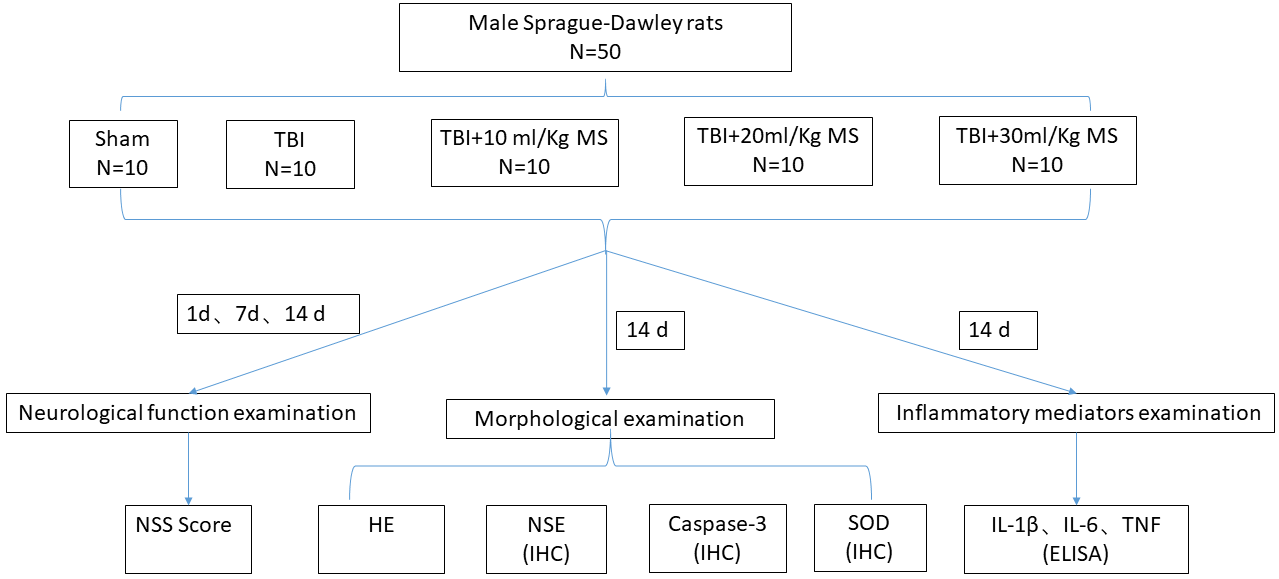


Supplement 2 Secondary part experimental protocol


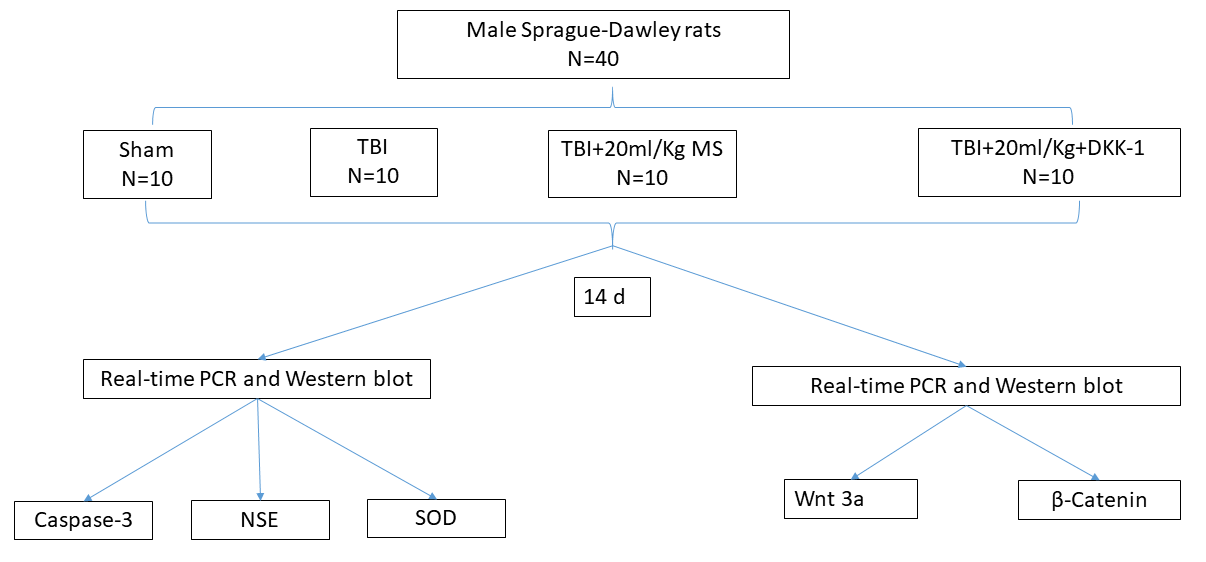

Supplement: Supplementary Materials — Supplement 1: first part—experimental protocol. Supplement 2: secondary part—experimental protocol. [file 3852450.f1.docx]
